# H9N2 Avian Influenza Virus Downregulates FcRY Expression in Chicken Macrophage Cell Line HD11 by Activating the JNK MAPK Pathway

**DOI:** 10.3390/ijms25052650

**Published:** 2024-02-24

**Authors:** Zhijian Sun, Wenjie Zhang, Jian Li, Kang Yang, Yanhao Zhang, Zili Li

**Affiliations:** 1 State Key Laboratory of Agricultural Microbiology, College of Veterinary Medicine, Huazhong Agricultural University, Wuhan 430070, China; szj@webmail.hzau.edu.cn (Z.S.);; 2 Key Laboratory of Preventive Veterinary Medicine in Hubei Province, Wuhan 430070, China

**Keywords:** FcRY, IgY, H9N2 avian influenza virus, chicken macrophage cell line, HD11 cells, c-jun N-terminal kinase

## Abstract

The H9N2 avian influenza virus causes reduced production performance and immunosuppression in chickens. The chicken yolk sac immunoglobulins (IgY) receptor (FcRY) transports from the yolk into the embryo, providing offspring with passive immunity to infection against common poultry pathogens. FcRY is expressed in many tissues/organs of the chicken; however, there are no reports investigating FcRY expression in chicken macrophage cells, and how H9N2-infected HD11 cells (a chicken macrophage-like cell line) regulate FcRY expression remains uninvestigated. This study used the H9N2 virus as a model pathogen to explore the regulation of FcRY expression in avian macrophages. FcRY was highly expressed in HD11 cells, as shown by reverse transcription polymerase chain reactions, and indirect immunofluorescence indicated that FcRY was widely expressed in HD11 cells. HD11 cells infected with live H9N2 virus exhibited downregulated FcRY expression. Transfection of eukaryotic expression plasmids encoding each viral protein of H9N2 into HD11 cells revealed that nonstructural protein (NS1) and matrix protein (M1) downregulated FcRY expression. In addition, the use of a c-jun N-terminal kinase (JNK) activator inhibited the expression of FcRY, while a JNK inhibitor antagonized the downregulation of FcRY expression by live H9N2 virus, NS1 and M1 proteins. Finally, a dual luciferase reporter system showed that both the M1 protein and the transcription factor c-jun inhibited FcRY expression at the transcriptional level. Taken together, the transcription factor c-jun was a negative regulator of FcRY, while the live H9N2 virus, NS1, and M1 proteins downregulated the FcRY expression through activating the JNK signaling pathway. This provides an experimental basis for a novel mechanism of immunosuppression in the H9N2 avian influenza virus.

## 1. Introduction

Yolk immunoglobulin (IgY) is produced by the mother and transmitted to the offspring, providing passive immunity against infection by common poultry pathogens until full maturation of the offspring’s autoimmune system [1]. The transfer of IgY involves two steps: the first is from the maternal circulation into the egg yolk of the oocyte, followed by the transfer from the egg yolk to the embryo, as mediated by FcRY [2]. FcRY consists of an ectodomain, a transmembrane domain, and an intracellular domain, and it has a molecular weight of approximately 180 kDa [3]. Its overexpression in polarized epithelial cells enables endocytosis, bidirectional transcytosis, and recycling [4]. FcRY is the only member of the mannose receptor (MR) family that functions as an Ig receptor [3]. FcRY has a signaling function, and secretory phospholipase A2 (sPLA2) combines with FcRY to produce intracellular signals that activate matrix metalloproteinase-9 (MMP-9) and induce cell death [5].

H9N2 is a low-pathogenic avian influenza virus (AIV) and one of the most widely circulating viruses in poultry that can pose a threat to humans by directly infecting or supplying the internal genes of multiple zoonotic avian influenza strains [6]. Host infection by the H9N2 virus causes great economic losses in the poultry industry owing to high mortality rates caused by immunosuppression or coinfection with other pathogens [7,8]. The H9N2 virus is an enveloped, single-stranded, negative-sense RNA virus with a genome consisting of eight segments encoding eleven core proteins, including hemagglutinin (HA), neuraminidase (NA), nucleoprotein (NP), matrix proteins (M1 and M2), nonstructural proteins (NS1 and NS2), and polymerase proteins (PA, PB1, PB2, and PB1-F2) [9]. NS1 protein binds and blocks IKKβ, thereby inhibiting the activation of nuclear factor kappa B (NF-κB) and the expression of antiviral genes [10,11]. The NS1 protein is an antagonist of viral and dsRNA-induced JNK mitogen-activated protein kinase (MAPK) activation [12]. PB2 protein blocks JAK1/STAT signaling by degrading JAK1, thereby impeding the production of interferon-stimulated genes (ISGs) [13]. The M1 protein of influenza viruses enhances viral pathogenicity by activating the Toll-like receptor 4 (TLR4) signaling pathway [14]. These studies suggest that the influenza virus suppresses host antiviral responses by modulating immune signaling through its core proteins.

MAPK cascade is an ancient and evolutionarily conserved innate immune signaling pathway that regulates numerous gene expressions involved in cell survival, proliferation, differentiation, and immune responses. This pathway mainly includes extracellular signal-regulated kinase (ERK), JNK, and p38 MAPK (p38) [15]. The MAPK signaling pathways are reportedly involved in influenza virus infection and play a crucial role in cellular protection against influenza viral infection [16,17]. Blockade of the JNK MAPK signaling pathway results in the inhibition of influenza virus-induced JNK and AP-1 (activator protein-1) activation and impairs IFN-β expression [18]. The AP-1 family consists of homodimers or heterodimers of jun (c-jun, junB, and junD), Fos (c-Fos, FosB, Fra-1, and Fra-2), and activating transcription factors (ATF-2 and ATF) [19]. AP-1 can bind to specific DNA sequences in the promoter or enhancer regions of target genes, thereby regulating the transcription of downstream target genes [18]. C-jun is the most extensively studied protein in the AP-1 complex. The pig neonatal Fc receptor (pFcRn) is a functional analog of FcRY in mammals that is regulated by JNK MAPK/c-jun [20].

The innate immune response is the first line of defense against viruses, and macrophages are important components of this system. Macrophages play several crucial roles in the immune system, including phagocytosis, pathogen suppression, and the secretion of cytokines and chemokines [21,22]. The innate immune response in chickens is mainly mediated by leukocytes, such as heterophiles and macrophages, which have important regulatory functions in host immune responses since they are dispersed in the body fluids and tissues of birds, producing various inflammatory mediators and cytokines [23]. For these cellular events, immune function will trigger specific signaling pathways, such as MAPK and NF-κB p65 [24,25]. The chicken macrophage cell line HD11 can be used as a model for H9N2 infection [26].

To date, research has mainly focused on FcRY structure and transport of IgY function, and no related studies have reported on the regulation of FcRY expression by pathogenic infection. Most members of the MR family are expressed by macrophages [27,28,29]. We speculated that FcRY was expressed in HD11 cells and used this cellular model to study the regulation of FcRY expression. Furthermore, we investigated whether FcRY expression was regulated by the MAPK pathway and the possible mechanisms involved.

## 2. Results

### 2.1. Susceptibility of HD11 Cells to H9N2 Virus Infection and Identification of FcRY Expression

**Figure 1:**
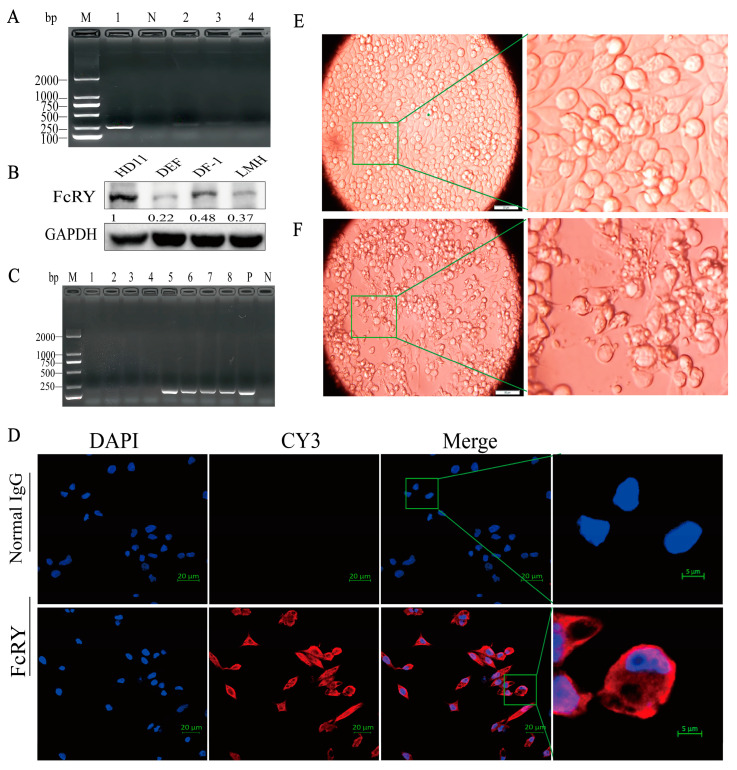
Susceptibility of HD11 cells to H9N2 virus infection and identification of FcRY expression. (**A**) Expression of FcRY in different cells was assessed by reverse transcription polymerase chain reaction (RT-PCR). Lane 1: HD11. Lane N: Negative, Lane 2: DEF, Lane 3: DF-1, Lane 4: LMH. Cells were harvested at the 24 h post inoculation. (**B**) Western blot analysis of FcRY expression in different cells. Relative FcRY protein levels were calculated by Image J 1.53t software and normalized to GAPDH. (**C**) Expression of the HA gene of the H9N2 virus at different times as assessed by RT-PCR. Lane M: Marker. Lanes 1–4: Control (6, 12, 18, and 24 h post-infection) uninfected HD11 cells. Lanes 5–8: H9N2 (6, 12, 18, and 24 h post-infection, respectively) infected HD11 cells. Lane P: Positive. Lane N: Negative. (**D**) Indirect immunofluorescence detection of FcRY expression in HD11 cells. Scale bar: 20 µm. HD11 cells were fixed, permeabilized, and blocked at 16 h post inoculation. (**E**) Control group. (**F**) The H9N2 virus infection group. Infection with the H9N2 virus at a multiplicity of infection (MOI) = 1 at 18 h. Scale bar: 50 µm.

We selected HD11, DEF (duck embryo fibroblast), DF-1(a continuous cell line of chicken embryo fibroblasts), and LMH (leghorn male hepatoma cell line) cell lines maintained in our laboratory to examine the intracellular expression of FcRY. FcRY was expressed in the HD11/DEF/DF-1/LMH cell lines, and the expression of FcRY in HD11 cells was higher than that in the other cell lines (Figure 1A,B). Therefore, HD11 cells were selected for subsequent experiments. Indirect immunofluorescence results further showed that FcRY can be expressed in HD11 cells (Figure 1D). Cytopathic effects (CPE) appeared in HD11 cells at 18 h post-infection (h.p.i.) after infection with the H9N2 virus at a multiplicity of infection (MOI) of one, including a reduction in cell numbers, cell shrinkage, aggregation, and the attachment of particles to the cell surface (Figure 1E,F). Viral genes were undetected in the control group, indicating that the H9N2 virus could infect and proliferate in HD11 cells (Figure 1C).

### 2.2. H9N2-Induced Infection Downregulated FcRY Expression in HD11 Cells

**Figure 2:**
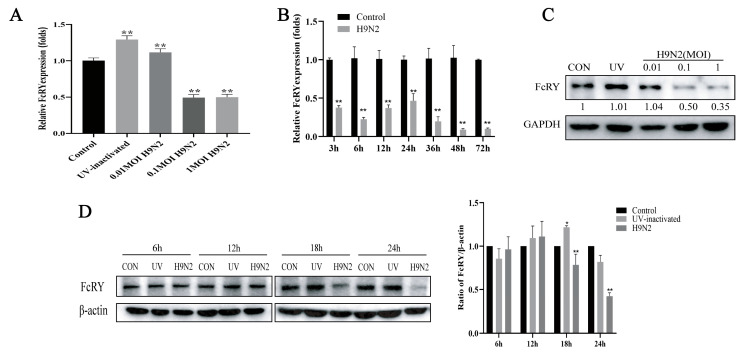
The effect of H9N2 virus infection on FcRY expression in HD11 cells. (**A**) RT-qPCR analysis of FcRY mRNA in HD11 cells by live H9N2 virus infection (MOI = 0.01, 0.1, 1) and UV-inactivation at 12 h. (**B**) RT-qPCR analysis of FcRY H9N2 virus mRNA in HD11 cells by infection (MOI = 0.1) for the indicated times. (**C**) HD11 cells were stimulated with the H9N2 virus (UV, MOI = 0.01, 0.1, and 1) for 18 h. Lysates of cells were used to test the FcRY protein expression level. (**D**) HD11 cells were stimulated with the H9N2 virus (MOI = 0.1) for the indicated times. The mRNA level of FcRY was standardized to β-actin. Relative FcRY protein levels were calculated by Image J 1.53t software and normalized to β-actin or GAPDH. All results were repeated at least three times, and data shown are means ± SEM (n = 3). * *p* < 0.05, ** *p* < 0.01 vs. the control group.

To determine whether the H9N2 virus is capable of modulating FcRY expression in HD11 cells, an infectious H9N2 virus or UV-inactivated virus was adsorbed onto HD11 cells at different MOIs, and FcRY mRNA levels were measured post-infection by quantitative reverse transcription polymerase chain reaction (RT-qPCR). FcRY mRNA levels were over two fold lower in the infection group at all time points post-infection compared to the control group (Figure 2A,B). However, treatment with UV-inactivated virus upregulated FcRY expression (Figure 2A,C). In addition, the protein level of FcRY decreased with the H9N2 virus dose (Figure 2C). The protein level of FcRY continued to decrease from 6 to 24 h after H9N2 virus stimulation, and the protein level at 18 h was significantly lower than that in the UV inactivated group and the control group. These results suggested that the H9N2 virus inhibited FcRY expression in HD11 cells in a time- and dose-dependent manner (Figure 2C,D). When HD11 cells were infected with MOI = 0.1 at 12 h, FcRY was significantly downregulated at the mRNA level. In combination with MOI = 1 (Figure 1E), obvious cytopathic changes and cell shedding occurred at 18 h post-infection. Therefore, to avoid excessive cell reactions, we ultimately chose MOI = 0.1 for subsequent experiments.

### 2.3. Role of MAPK Pathways in the Downregulation of FcRY in HD11 Cells by H9N2 Virus

**Figure 3:**
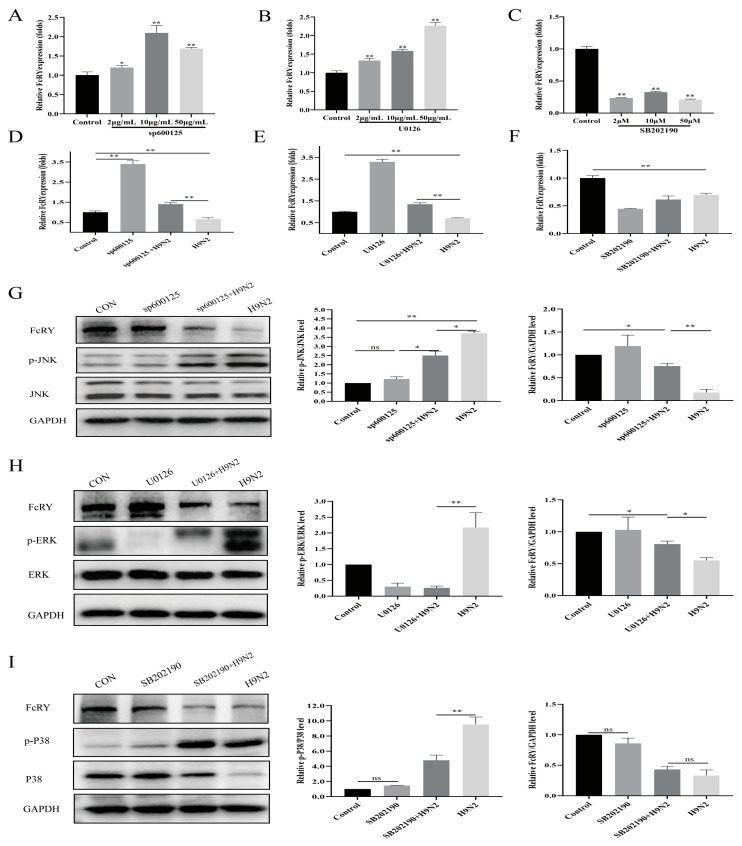
The effect of the MAPK signaling pathway on FcRY expression. (**A**–**C**) HD11 cells were continuously treated with the indicated doses of sp600125, U0126, and SB202190 for 12 h. (**D**–**F**) HD11 cells were pre-treated with sp600125 (10 µg/mL), U0126 (20 µg/mL), and SB202190 (20 µM) for 1 h, followed by incubation with the H9N2 virus (MOI = 0.1) for 12 h, respectively. FcRY mRNA levels were analyzed by RT-qPCR. The relative mRNA levels were normalized to the corresponding β-actin mRNA levels. * *p* < 0.05, ** *p* < 0.01. (**G**–**I**) The expression of FcRY and the ratio of p-JNK/JNK, p-ERK/ERK, p-p38/p38 in HD11 cells using the following inhibitors: sp600125 (10 µg/mL), U0126 (20 µg/mL), and SB202190 (20 µM), respectively. GAPDH was used as a loading control. The right panel represents protein band quantification determined by densitometry and normalized to GAPDH. All results were repeated at least three times, and data shown are means ± SEM (n = 3). * *p* < 0.05, ** *p* < 0.01, ns: not significant.

This study used MAPK inhibitors to elucidate the role of the MAPK signaling pathway on FcRY expression. We selected sp600125, U0126, and SB202190 as JNK, ERK, and p38 inhibitors, respectively, among the numerous MAPK inhibitors reported to act on MAPK. The FcRY mRNA levels in HD11 cells significantly increased in a dose-dependent manner after administering JNK and ERK inhibitors (Figure 3A–C). Furthermore, JNK and ERK inhibitors resisted FcRY downregulation by the H9N2 virus at the mRNA level (Figure 3D,E). The expression levels of FcRY in HD11 cells were significantly reduced after treatment with the p38 inhibitor (Figure 3F). MAPK inhibitors reduced the ratios of p-JNK/JNK, p-p38/p38, and p-ERK/ERK. The JNK and ERK inhibitors antagonized FcRY downregulation by the H9N2 virus at the protein level (Figure 3G,H). The p38 inhibitor did not affect FcRY downregulation by H9N2 (Figure 3I). These results indicated that the MAPK signaling pathway is involved in regulating FcRY expression by the H9N2 virus.

### 2.4. Construction of Eukaryotic Expression Plasmids for H9N2 Viral Gene Segments and Their Effect on FcRY Expression

**Figure 4:**
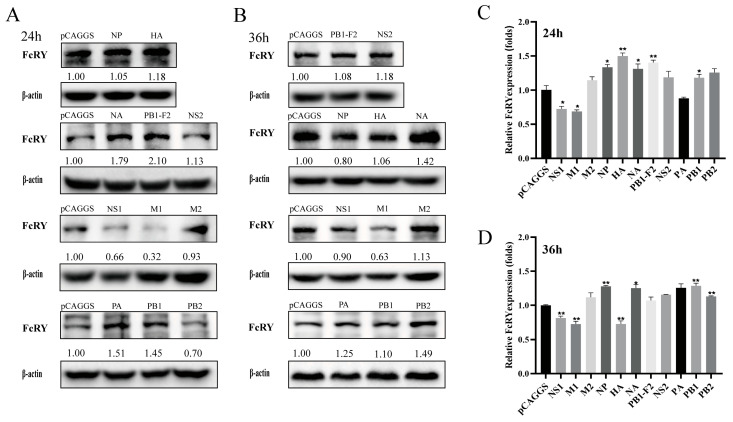
The effect of eukaryotic expression plasmids for each gene segment of the H9N2 virus on FcRY. (**A**,**B**) HD11 cells were transiently transfected with the constructed eukaryotic expression plasmids of H9N2 viral genes. Cells were harvested 24 and 36 h after transfection, and protein extracts were prepared for western blotting. Protein bands determined by densitometry were quantified and normalized to β-actin. (**C**,**D**) The same transfection operation as the previous step was followed, and the cell samples were harvested 24 and 36 h after transfection to extract RNA, as well as to measure the mRNA expression. FcRY mRNA levels were analyzed by RT-qPCR, and the relative mRNA levels were normalized to the corresponding β-actin mRNA levels. * *p* < 0.05, ** *p* < 0.01. Error bars represent the mean ± SEM (n = 3).

We constructed a series of eukaryotic expression plasmids containing H9N2 virus genes to further explore the effect of H9N2 viral proteins on FcRY expression. The plasmids were transfected into HD11 cells for 24 h and 36 h, respectively. The results showed that NS1, M1 proteins could downregulate the expression of FcRY. In contrast, PA, NA, PB1, NS2 proteins could upregulate the expression of FcRY. PB1-F2, HA proteins only increased the expression of FcRY at 24 h. M2, PB2 proteins only upregulated FcRY expression at 36 h. NP protein showed inconsistent regulation of FcRY at the mRNA and protein levels at 36 h. Different viral gene segments had different effects on FcRY expression at different time points (Figure 4A–D). NS1 and M1 downregulated FcRY expression at the mRNA and protein levels.

### 2.5. NS1 and M1 Downregulate FcRY Expression through the JNK MAPK Pathway

**Figure 5:**
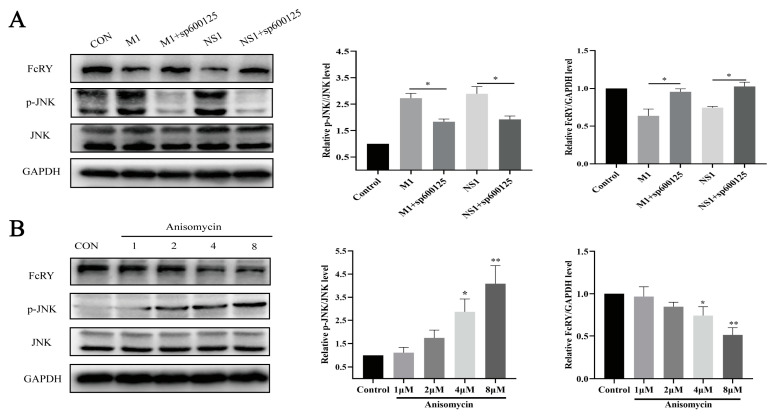
The JNK MAPK pathway regulates FcRY expression. (**A**) HD11 cells were used to inhibit pretreatment for 1 h, while HD11 cells were transfected by the indicated plasmids for 24 h, followed by examining the protein levels by western blotting. The right panel represents protein band quantification determined by densitometry and normalized to GAPDH, * *p* < 0.05. (**B**) HD11 cells were treated with Anisomycin at the indicated concentrations for 24 h. HD11 cells were harvested for protein analysis. The right panel represents protein band quantification determined by densitometry and normalized to GAPDH, * *p* < 0.05, ** *p* < 0.01.

These studies showed that the JNK MAPK pathway is involved in the downregulation of FcRY by live H9N2 viruses. We pretreated HD11 cells with sp600125 (a JNK inhibitor) for 1 h and transfected them with NS1 and M1 eukaryotic expression plasmids for 24 h to verify whether NS1 and M1 proteins would regulate the expression of FcRY through the JNK MAPK pathway. The p-JNK/JNK ratio significantly decreased after inhibitor treatment. Simultaneously, the FcRY levels were restored (Figure 5A). In addition, we chose 1, 2, 4, and 8 μM doses of JNK activators to verify the regulation of the JNK MAPK pathway on FcRY (Figure 5B). The ratio of p-JNK/JNK increased as the dose increased, whereas FcRY expression decreased. These results indicated that NS1 and M1 reduced FcRY expression by activating the JNK MAPK pathway.

### 2.6. Construction of FcRY Promoter Luciferase Report Plasmids

**Figure 6:**
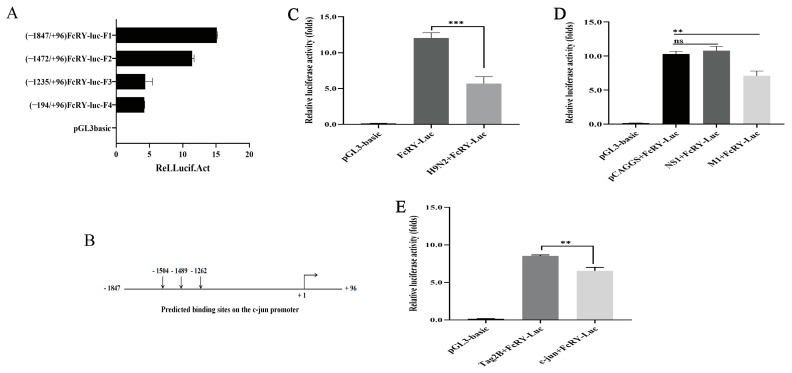
Construction of FcRY promoter luciferase report plasmids. (**A**) Basal promoter activity of the FcRY-luc-F (1–4) in HD11 cells. (**B**) Predicted transcription factor binding site of c-jun in the primary active region (−1847/−1235). (**C**) HD11 cells were co-transfected with FcRY promoter luciferase report plasmids (−1847/+96, F1) and a Renilla luciferase vector (pRL-TK-luc). Cells were subsequently treated with the H9N2 virus (MOI = 0.1) for 12 h and harvested for luciferase assays. *** *p* < 0.001. (**D**,**E**) NS1, M1, and c-jun eukaryotic expression plasmids were co-transfected in HD11 cells with FcRY promoter constructs (−1847/+96, F1) and a luciferase reporter vector (pRL-TK-luc), respectively. Luciferase activity was measured 36 h post-transfection. ** *p* < 0.01, ns: not significant.

Promotor constructs with different 5’-ends were generated to further identify the critical region for the transcriptional activity of FcRY. We created four luciferase reporter gene constructs with sequentially shortened fragments of the promoter region: FcRY-luc-F1 (−1847/+96), FcRY-luc-F2 (−1472/+96), FcRY-luc-F3 (−1235/+96), and FcRY-luc-F4 (−194/+96). Transfection of the four luciferase reporter plasmids into HD11 cells significantly increased basal promoter activity compared to that of the empty vector. The promoter activities of the F3 and F4 luciferase reporter plasmids were significantly lower than those of the other two plasmids (Figure 6A). These data revealed that the region between −1847 and −1235 was required to activate the FcRY promoter. Subsequently, we selected the highest luciferase activity of FcRY-luc-F1 (−1847/+96) to test whether the FcRY promoter was affected by the live H9N2 virus, NS1 protein, and M1 protein. FcRY promoter activity was downregulated in HD11 cells by live H9N2 virus infection or M1 overexpression plasmid transfection (Figure 6C,D). Multiple potential binding sites for the transcription factor c-jun were predicted between −1847 and −1235 using the online tool JASPAR (Figure 6B). To assess the involvement of c-jun on FcRY expression, we investigated whether overexpression of c-jun affects the promoter activity of FcRY. The result indicated that the overexpression of c-jun downregulates FcRY expression (Figure 6E).

## 3. Discussion

FcRY was first discovered and cloned over 20 years ago. Research has mainly focused on the structure, function, and mechanisms of IgY transport, but no studies have reported on the regulation of FcRY expression. FcRY RNA expression is observed in the yolk sac membrane, liver, ovary, and oviduct spleen, according to northern blot analysis [3]. We found that FcRY was expressed in a wide variety of cells according to RT-PCR and western blot assays, with the highest levels observed in HD11 cells using several poultry cell lines maintained in our laboratory (Figure 1A,B). Further indirect immunofluorescence experiments showed that FcRY was widely expressed in HD11 cells (Figure 1D). These findings indicate that FcRY is widely distributed within cells, which correlates with the MR family distribution characteristics reported in the literature, indicating that 10–30% of the receptors are located at the cell surface and 70–90% are located intracellularly at the steady state [27]. The H9N2 virus can infect HD11 cells and adversely affect the host’s immune response [26]. Efficient influenza virus replication reduces macrophage phagocytosis, presumably by reducing cell surface receptor expression [30]. There is evidence that influenza A virus PR8 infection significantly reduces the mRNA levels of some of these receptors in macrophages, including C-type lectin domain family 7 member A (CLEC7A), macrophage scavenger receptor 1 (MSR1), and CD36 [31]. Alternatively, activated bone marrow-derived macrophages (BMDMs) infected with the influenza virus, downregulated their surface MR (CD206) expression, altering the inflammatory phenotype of macrophages [32]. This illustrates that macrophages play an important role in innate immunity and that influenza virus infection may impair their ability to clear pathogens. We similarly found that the H9N2 virus downregulated FcRY mRNA and protein levels in HD11 cells in a dose-dependent manner (Figure 2A,C), possibly leading to a decrease in the ability of macrophages to defend against viruses after H9N2 infection, thereby suppressing the immune function of the body. The experiment offers a new perspective for understanding the impact of H9N2 virus on macrophages and provides a theoretical foundation for further studying other viruses that infect macrophages. Nevertheless, caution is still necessary when generalizing the findings to all viruses that infect macrophages. Biological differences between viruses are significant, and different viruses may have distinct mechanisms and degrees of influence on macrophages. Nevertheless, the H9N2 avian influenza virus can provide internal gene segments to other subtypes of avian influenza viruses, enabling the generation of new genotypes [33]. This internal gene connection enables future research to explore the mechanism of interaction between other avian influenza virus subtypes and macrophages, enabling a comprehensive understanding of the universality of these findings and their applicability during different virus infections.

This study found that H9N2 significantly increased the levels of phosphorylated p38 MAPK, ERK, and JNK, indicating that H9N2 activated the p38, ERK, and JNK signaling pathways, consistent with the results of previous studies [34]. After activation, MAPKs regulate the upregulation or downregulation of many genes. This study showed that FcRY expression was downregulated after H9N2 infection, and we speculated that FcRY expression was regulated by the MAPK pathway. We found that JNK and ERK inhibitors significantly elevated the FcRY expression level and antagonized the downregulation of FcRY by the H9N2 virus (Figure 3). JNK activators decreased FcRY expression in a dose-dependent manner (Figure 5). This indicated that the JNK MAPK signaling pathway regulated FcRY expression. It is well-documented that c-jun (a downstream component of the JNK MAPK signaling pathway and the most extensively studied protein in the AP-1 complex) may be involved in the development of viral infection [35]. We constructed a full-length FcRY promoter region (−1847/+96) fluorescent reporter plasmid and found that the main active region was −1847 bp to −1235 bp by truncation experiments. Bioinformatics analysis revealed the presence of multiple potential c-jun/AP-1 binding sites in this fragment (Figure 6). Subsequently, a dual fluorescence reporter assay using the FcRY-luc-F1 plasmid (−1847/+96) with the highest basal promoter activity showed decreased promoter activity in the FcRY luciferase reporter assay after overexpression of the c-jun eukaryotic plasmid, indicating that c-jun downregulates FcRY gene transcript expression. AP-1 is generally defined as a transcriptional activator; however, it is also a transcriptional repressor [36,37]. Takakura et al. found that c-jun could inhibit telomerase directly by inhibiting the transcription of human cell telomerase reverse transcriptase (hTERT) gene promoter [38]. C-jun can also act indirectly to repress the expression of genes by suppressing other promoter activities [39]. Our results demonstrate that overexpression of the c-jun transcription factor downregulates FcRY transcription and is a negative regulator of FcRY expression.

We transfected eukaryotic expression vectors encoding different viral proteins of H9N2 into HD11 cells to identify viral proteins that downregulate FcRY expression in HD11 cells. Different viral proteins differentially affected FcRY expression at different time points (Figure 4). NS1 and M1 consistently downregulated FcRY expression, whereas NA and PA upregulated FcRY expression. PB1-F2 protein upregulated FcRY expression at 24 h and had no significant effect on FcRY after 36 h. Combined with the results that live H9N2 virus downregulated FcRY, we further investigated the mechanism by which NS1 and M1 proteins downregulated FcRY expression. The NS1 protein is one of the first viral proteins to be expressed during influenza virus infection of host cells and is a major pathogenic factor that can modulate host immune responses by acting on signaling pathways [40]. NS1 overexpression activates the JNK MAPK signaling pathway in certain influenza virus subtypes [41]. The M1 protein of the influenza virus induces apoptosis by activating the TLR4 signaling pathway [14]. Similarly, we found that the overexpression of either influenza NS1 or M1 proteins activated the JNK MAPK signaling pathway, causing a decrease in FcRY expression. A dual-luciferase reporter plasmid showed that the M1 protein downregulated *FcRY* expression at the transcriptional level, which was consistent with the overexpression of the transcription factor c-jun. It has been illustrated that M1 protein downregulates the expression of FcRY by activating transcription factor c-jun. Previous studies show a fourfold increase in RNA-binding proteins (AUF1), which destabilizes target mRNA in JNK-activated cells [42], and JNK plays an important role in regulating mRNA stability [43]. Therefore, we speculated that NS1 protein downregulates FcRY expression by decreasing the stability of post-transcriptional FcRY mRNA. In summary, NS1 and M1 proteins use different methods to downregulate FcRY expression after activating the JNK MAPK pathway in HD11 cells. These results may offer experimental support for a potential new mechanism of immunosuppression caused by the H9N2 avian influenza virus.

## 4. Materials and Methods

### 4.1. Cells and Virus

HD11 cells (generously donated by Dr. Tengfei Wang of the Key Laboratory of Agricultural Animal Genetics, Breeding and Reproduction, Ministry of Education, College of Animal Science and Technology and College of Veterinary Medicine, Huazhong Agricultural University, Wuhan, China) were cultured in RPMI-1640 medium (Hyclone, Logan, UT, USA) supplemented with 10% fetal bovine serum (Gibco, Waltham, MA, USA) and 1% penicillin/streptomycin in an atmosphere of 5% CO_2_ at 37.5 °C. DF-1 cells (obtained from the China Center for Type Culture Collection, Wuhan, China), LMH cells (kindly provided by Dr. Meng Liu of the Key Laboratory of Animal Embryo Engineering and Molecular Breeding of Hubei Province, Huazhong Agricultural University, Wuhan, China), and DEF cells (kindly provided by Dr. Sen Li of the State Key Laboratory of Agricultural Microbiology, College of Veterinary Medicine, Huazhong Agricultural University, Wuhan, China). The above three cells were grown in DMEM (Hyclone, Logan, UT, USA supplemented with 10% FBS, 100 μg/mL penicillin/streptomycin in a humidified atmosphere containing 5% CO_2_ at 37 °C. The H9N2 viral strain (A/chicken/Hubei/01/1999, Accession numbers: CY077081-CY077090) was isolated from a chicken infected with AIV in the Hubei Province. It was maintained in our laboratory (the State Key Laboratory of Agricultural Microbiology, College of Veterinary Medicine, Huazhong Agricultural University, Wuhan, China) and stored at −80 °C until it was used.

### 4.2. Plasmids, Antibodies, and Reagents

All H9N2 virus genes were individually cloned into the mammalian expression vector pCAGGS (Addgene, Cambridge, MA, USA, kept in our laboratory). Sequences of primers used for constructing plasmids are listed in Table 1. Full length c-jun was cloned into the pCMV-Tag2B-Flag plasmid (Addgene, Cambridge, MA, USA, kept in our laboratory) using homologous recombination. Luciferase reporter plasmids pGL3 and pRL-TK were purchased from Promega (Madison, WI, USA). Horseradish peroxidase-conjugated goat anti-rabbit or anti-mouse IgG, mouse monoclonal anti-glyceraldehyde 3-phosphate dehydrogenase (GAPDH) antibody, and rabbit monoclonal anti-β-actin antibody were purchased from ABclonal (ABclonal, Wuhan, China). Rabbit monoclonal antibodies against phospho-ERK1/2, ERK1/2, phospho-p38, p38, phospho-JNK1/2/3, and JNK1/2/3 were purchased from HuaAn (Huabio, Hangzhou, China). The FcRY monoclonal antibody was prepared in our laboratory, while the FcRY polyclonal antibody was prepared by Wuhan Daian Biotech, Ltd. (Daian, Wuhan, China).

### 4.3. RNA Extraction and RT-qPCR Analysis

HD11s were harvested at the indicated time points post stimulation, while the total RNA was isolated using TRIzol reagent (Invitrogen, Carlsbad, CA, USA). Subsequently, 1 μg of total RNA from each sample was reverse-transcribed into cDNA with an iScript cDNA synthesis kit (Bio-Rad, Hercules, CA, USA). The cDNA was used to perform the RT-qPCR with SYBR Green Supermix (Bio-Rad) following the manufacturer’s instructions. Measured transcripts were normalized to the chicken β-actin mRNA level. The sequences of the primers used in this experiment are shown in Table 1. H9N2 virus detection primer synthesis was performed as previously described [44]. The detection gene is part of HA, and the amplified fragment length was 140 bp.

### 4.4. Western Blotting

HD11 cells were cultured to 70–80% confluence in 6-well plates and stimulated with the H9N2 virus or a eukaryotic expression plasmid. The total protein in the cells was extracted using a RIPA lysis buffer (P0013B, Beyotime, Wuhan, China) supplemented with a protease inhibitor cocktail (Roche, San Mateo, CA, USA). A western blot analysis was performed, as previously described [45].

### 4.5. Construction of Reporter Plasmids

Chicken genomic DNA was isolated from HD11 cells using the TIANamp Genomic DNA Kit (TIANGEN, Beijing, China). A 1943 bp (−1847/+96) fragment containing the 5′-flanking region of the chicken FcRY was amplified with primers according to the HD11 cell sequence (NM_206983.2) (Table 1). Polymerase chain reaction amplification was performed using KOD-Plus-Neo DNA polymerase (KOD-401, Toyobo, Osaka, Japan), while the products were subcloned into plasmid pGL3.0 to generate the FcRY reporter vector F1 (−1847/+96). The truncated fragments of the FcRY promoter were amplified with the indicated primers shown in Table 1 and subcloned into pGL3 with the same method to construct the deletion mutant plasmids F2 (−1472/+96), F3 (−1235/+96), and F4 (−194/+96).

### 4.6. Plasmid Transfection and Luciferase Assay

HD11 cells (1 × 10^5^ cells /well) were seeded in 24-well plates and transfected with FcRY reporter vectors (980 ng/well) and pRL-TK (20 ng/well) using EZ Trans (Life-iLab, Shanghai, China) for 24 h. Cells were subsequently treated with the H9N2 virus (MOI = 0.1) for 12 h. For overexpression experiments, pCAGGS-NS1 (490 ng/well), pCAGGS-M1 (490 ng/well), pCMV-Tag2B (490 ng/well), and pCMV-Tag2B-c-jun (490 ng/well) were transfected with FcRY luciferase reporter plasmid (−1847/+96, F1, 490 ng/well) and Renilla luciferase pRL-TK vector (20 ng/well) into HD11 cells with EZ Trans, respectively, and the empty vector was used as a control. Cells were harvested at the indicated times, and luciferase activity was measured using a Dual-Luciferase Assay System (Promega, Fitchburg, MA, USA). Firefly luciferase values were normalized to those for Renilla luciferase activity.

### 4.7. Indirect Immunofluorescence

HD11 cells were cultured to 10–20% confluence on sterilized coverslips. After 16 h, the cells were fixed, permeabilized, and blocked, as previously described [46]. Subsequently, cells were incubated with a primary antibody, AlexaFluor-conjugated secondary antibody, and finally stained with 4′-6-diamidino-2-phenylindole (DAPI) (Invitrogen, Carlsbad, CA, USA). Signals were collected at 100× magnification using a Zeiss LSM 510 Meta-confocal microscope (Carl Zeiss, Oberkochen, Germany).

### 4.8. Statistics and Data Analysis

Data are presented as the mean standard error of the mean (SEM). All experiments were independently performed at least three times, and statistical significance was determined by one-way analysis of variance (ANOVA), followed by Dunnett’s post hoc test using standard Prism 8.3.0 (GraphPad Software, San Diego, CA, USA) software. A *p*-value of <0.05 and <0.01 was considered significant.

## 5. Conclusions

In conclusion, FcRY was expressed at high levels in HD11 cells and distributed in the cytosol and cell membrane. The main active region of the FcRY promoter was between −1847 and −1235. The M1 protein repressed FcRY expression at the transcriptional level. Furthermore, c-jun negatively regulated FcRY expression. The live H9N2 virus, NS1, and M1 proteins downregulated FcRY expression by activating the JNK MAPK signaling pathway.

## Figures and Tables

**Table 1:** Primers used in the experiments.

Target	Forward Primer (5′-3′)	Reverse Primer (5′-3′)
β-actin	TTGTTGACAATGGCTCCGGT	TCTGGGCTTCATCACCAACG
H9N2	AATGTYCCTGTGACACATGCCAA	AGRTCACAAGAAGGRTTGCCATA
FcRY	GCTACACAAACTGGAACAAGCA	CTTCTGACTCCCACCCAAAGT
FcRY-luc-F1	GGGGTACCGTGTTCAGCAGTTGGACGT	GAAGATCTCGCCGCGGCTTGGCACGAC
FcRY-luc-F2	GGGGTACCAGGGTCCTCGAAAAACAGATCATC	GAAGATCTCGCCGCGGCTTGGCACGAC
FcRY-luc-F3	GGGGTACCAGAGCTGCCCAGAATAAGCAAGTTGC	GAAGATCTCGCCGCGGCTTGGCACGAC
FcRY-luc-F4	GGGGTACCAGCCGTTCCTCCCCCCGAG	GAAGATCTCGCCGCGGCTTGGCACGAC
pCMV-c-jun	CAGGAATTCGATATCAAGCTTATGAGTGCAAAGATGGAGC	GGTACCGGGCCCCCCCTCGAGTCAAAACGTTTGCAACTGT
pCAGGS-NP	CGGAATTCATGGCGCTTCAAGGCACC	CCTCGAGTTAATTGTCATACTCCTCTGCATTGTC
pCAGGS-HA	CGAGCTCATGGAAGTAGTATCACTA	GCTCGAGTTATATACAAATGTTGCATCTGC
pCAGGS-NA	GAGCTCATGAATCCAAATCAGAAGATAATAGC	CCTCGAGTTATATAGGCATGA AGTTGATATTCGC
pCAGGS-PB1-F2	CCGGAATTCATGGATCCA AACACTGTGTC	CCTCGAGCTATTTTGGAGAGAGTGGAGG
pCAGGS-NS2	CGAATTCATGGATCCAAACACTGTGTCAAGCTT	GCTCGAGTTAAATAAGCTGAAACGAGAAAGTTC
pCAGGS-M1	CGAATTCATGAGCCTTCTAACCGAGG	GCTCGAGTCACTTGAATCGCTGCATC
pCAGGS-M2	GGAATTCATGAGCCTTCTAACCGAGGTCGAAAC	GCTCGAGTTACTCCAGCTCTATGTTGAC
pCAGGS-PA	TTCCAGATTACGCTGAATTCATGGAAGATTTTGT	TTAAGATCTGCTAGCTCACGCTATTTCAGTGCAT
pCAGGS-PB1	GGCATGCCCGGGTACCATGGATGTCAATCCGA	TGTTCCATGGCTGTATGGGGGATCTCCAGTATAA
pCAGGS-PB2	AGAGCTAATGTCGCAATCCCGCACCCGCGAGAT	AACACTACTTGTCCCGCCTGATACTGGTAGGAA
pCAGGS-NS1	GGAATTCATGGATCCAAACACTGTGTCAAGC	CCTCGAGCTATTTTGGAGAGAGTGGAGGTCT
pCAGGS-M1	CGAATTCATGAGCCTTCTAACCGAGG	GCTCGAGTCACTTGAATCGCTGCATC

## Data Availability

The data that support the findings of this study are available from the corresponding author upon reasonable request.
